# Analysis of characteristics related to interstitial lung disease or pulmonary hypertension in patients with dermatomyositis

**DOI:** 10.1111/crj.13720

**Published:** 2023-11-20

**Authors:** Chulin Wang, Han Yang, Tongfen Li, Yiqiong Wen, Heran Yang, Weifeng Tang, Lin Li, Shibo Sun

**Affiliations:** ^1^ Department of Pulmonary and Critical Care Medicine, First Affiliated Hospital Kunming Medical University Kunming China; ^2^ Department of Anesthesiology Kunming Medical University Kunming China; ^3^ Department of Clinical Medicine Kunming Medical University Kunming China

**Keywords:** anti‐melanoma differentiation‐associated gene 5 antibodies, dermatomyositis, interstitial lung disease, pulmonary hypertension

## Abstract

**Introduction:**

Dermatomyositis (DM) is often associated with interstitial lung disease (ILD) or pulmonary hypertension (PH). The aim of this study was to investigate the clinical characteristics of DM patients with ILD or PH.

**Methods:**

This study retrospectively analysed the clinical characteristics of 372 patients with DM, including cytokines, lymphocyte subsets, immunoglobulin and complement. The DM patients were divided into different groups according to whether complicated with ILD, PH or anti‐melanoma differentiation‐associated gene 5 antibodies (MDA5). A qualitative and quantitative data analysis was performed.

**Results:**

IgG, IgA and IgM in the DM‐associated ILD (ILD‐DM) were higher than that of the DM non‐complicating ILD (Non‐ILD‐DM) (*p* = 0.022, 0.002 and 0.029, respectively). Meanwhile, IL‐6 (*p* = 0.008) and IL‐10 (*p* = 0.001) were increased in the DM‐associated PH (PH‐DM) than in the DM non‐complicating PH (Non‐PH‐DM), while IL‐17 (*p* = 0.004), double positive (DP) cell ratio and B lymphocyte ratio were reduced in the PH‐DM. Moreover, the incidence of ILD and levels of C4 were higher in the DM with MDA5 (MDA5^+^ DM) than that of the DM without MDA5.

**Conclusion:**

ILD‐DM has higher IgG, IgA and IgM than that of Non‐ILD‐DM. PH‐DM has higher IL‐6, IL‐10 and lower IL‐17, DP cell ratio and B lymphocyte ratio than that of Non‐PH‐DM.

## Graphical abstract

A comparison of ILD‐DM and Non‐ILD‐DM revealed significant differences in IgG, IgA and IgM. Meanwhile, significant differences in IL‐6, IL‐10, IL‐17, DP cell ratio and B lymphocyte ratio found when comparing PH‐DM and Non‐PH‐DM.

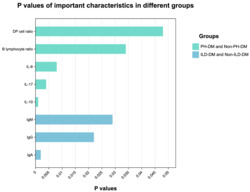

## 1 INTRODUCTION

Dermatomyositis (DM) is an idiopathic inflammatory disease that is frequently accompanied by inflammation, immune‐mediated organ damage.^1^ DM causes muscle, skin, joints, heart and lung damage,^1^ among which lung injury predominantly presents interstitial lung disease (ILD) and pulmonary hypertension (PH).^2^ ILD is a group of common heterogeneous diseases characterized by fibrosis or inflammation in the interstitial space.^3^, ^4^ As the most serious complication of DM, ILD is mainly caused by impaired the exchange of gas leading to respiratory distress, which significantly increases the mortality rate of DM.^4^, ^5^ PH is a pulmonary vascular disease characterized by a variety of aetiologies,^3^ which are linked to excessive vasoconstriction, vascular remodelling, inflammation and thrombosis in situ.^6^ Similarly, PH is closely related to DM complications and impairs DM prognosis.^7^ However, few researches on which DM are more likely to develop PH have been found so far. Increasing researches suggest that immune cells and inflammatory cytokines play a crucial role in PH and ILD.^4^, ^8^ Accordingly, this study retrospectively analysed the clinical characteristics of 372 patients with DM and compared the inflammatory, immunization and demographic characteristics of DM with ILD or PH.

## 2 METHODS

### 2.1 Patients

Patients with DM admitted to the First Affiliated Hospital of Kunming Medical University between August 2015 and September 2022 were included. DM was diagnosed according to the criteria proposed by Bohan and Peter in 1975.^9^ The following indicators for analysis were included, demographic characteristics (age, gender, ethnicity, smoking, drinking and family history), clinical data (diagnosis), relevant laboratory tests (cytokines, lymphocyte subsets, immunoglobulins and complement) and imaging manifestations (echocardiography and pulmonary CT).

### 2.2 Comparison of patients' characteristics in different situations

The subjects were grouped according to different characteristics. According to Chinese ethnicity, all patients were divided into two groups, the Han population patients with DM and the ethnic minority patients with DM. According to the presence or absence of ILD, all patients were divided into two groups, the DM‐associated ILD (ILD‐DM) and the DM non‐complicating ILD (Non‐ILD‐DM). ILD was diagnosed on the basis of clinical and physical examination results and imaging features.^1^ According to the presence or absence of PH, all patients were divided into two groups, the DM‐associated PH (PH‐DM) and the DM non‐complicating PH (Non‐PH‐DM). PH was defined as a pulmonary artery systolic pressure (PASP) ≥ 30 mmHg at rest, measured by Doppler echocardiography.^10^ In addition, we conducted subgroup analyses of DM patients with or without ILD. According to the presence or absence of PH, all the ILD‐DM patients were divided into two groups (PH/Non‐PH) and all the Non‐ILD‐DM patients were divided into two groups (PH/Non‐PH). Finally, according to the presence or absence of anti‐melanoma differentiation‐associated gene 5 antibodies (MDA5), all patients were divided into two groups, the DM with MDA5 (MDA5^+^ DM) and the DM without MDA5 (MDA5^−^ DM). MDA5^+^ was determined using an anti‐MDA5 antibody detection method according to a linear immunoassay (Euroimmun [Germany] or D‐Tek [Belgium]).^11^ Differences in demographic characteristics, cytokines, lymphocyte subsets, immunoglobulins and complement were compared between variable subgroups of patients.

### 2.3 Statistical analysis

Analysis was performed using SPSS 26.0. The chi‐square test was used to compare the categorical variables. As for the continuous variables, the Shapiro–Wilk test was first performed to determine whether the data were normally distributed. If the data were normally distributed, the independent samples *t*‐test was employed to compare it, and the data were presented as mean ± standard deviation (SD). If the data were not normally distributed, the Mann–Whitney U test was employed, and the data were presented as the median (25th percentile, 75th percentile).

## 3 RESULTS

### 3.1 Baseline characteristics

A total of 372 patients with DM was included in this study. The demographic characteristics were listed in Table 1.

**TABLE 1:** Baseline characteristics (patients with DM, *n* = 372).

Characteristics	Values
Age (years)	
0–17	19 (5.11)
18–65	313 (84.14)
66–79	34 (9.14)
80–99	6 (1.61)
Gender	
Male	124 (33.33)
Female	248 (66.67)
Ethnicity	
Han	320 (86.02)
Minority	52 (13.98)
Smoking	64 (17.20)
Drinking	41 (11.02)
Family history	3 (0.81)
ILD	158 (42.47)
PE	3 (0.81)
PH	85 (22.85)
MDA5^+^	30 (8.06)

*Note*: Values are *n* (%) except where indicated.

Abbreviations: DM, dermatomyositis; ILD, interstitial lung disease; MDA5, anti‐melanoma differentiation‐associated gene 5 antibodies; PE, pulmonary embolism; PH, pulmonary hypertension.

### 3.2 DM patients in different ethnic groups in China

The Han population was significantly older than that of the ethnic minority DM patients (*p* < 0.001). In addition, IL‐8 levels in DM patients were decreased in the Han population (median [IQR]; 16.00 [10.00–22.25] pg/mL) than in the ethnic minority (median [IQR]; 22.00 [16.00–55.00] pg/mL (*p* = 0.038). Also, platelet (PLT) and plateletcrit (PCT) were significantly lower in the Han population than in the ethnic minority DM patients (*p* < 0.001 and 0.001, respectively). Meanwhile, cytotoxic/suppressor T‐cell (Tc/Ts) counts and IgM levels were significantly lower in the Han population than in the ethnic minority DM patients (*p* = 0.013 and 0.006, respectively) (Table 2).

**TABLE 2:** DM patients in different ethnic groups in China.

	Han (*n* = 320)	Minority (*n* = 52)	*p* value
Age (years)	50 (42, 59)	44 (33.25, 51)	0.000^#^
Gender (male/female)	109/211	15/37	0.459
Smoking (yes/no)	263/57	45/7	0.441
Drinking (yes/no)	33/287	8/44	0.279
Family history (yes/no)	3/317	0/52	1.000
ILD (yes/no)	137/183	21/31	0.743
PE (yes/no)	3/317	0/52	1.000
PH (yes/no)	78/103	7/17	0.193
MDA5 (+/−)	27/293	3/49	0.703
PASP (mmHg)	28.00 (21.00, 34.00)	23.00 (21.00, 31.50)	0.190
IL‐1β (pg/mL)	1.18 (0.60, 4.73)	0.64 (0.38, 2.46)	0.290
IL‐2 (pg/mL)	1.17 (0.58, 2.07)	1.41 (0.59, 2.20)	0.775
IL‐4 (pg/mL)	1.02 (0.53, 1.65)	1.12 (0.46, 1.74)	0.782
IL‐5 (pg/mL)	1.86 (0.93, 3.49)	2.19 (0.97, 2.46)	0.816
IL‐12P70 (pg/mL)	0.93 (0.49, 1.69)	0.91 (0.23, 1.78)	0.647
IL‐17 (pg/mL)	1.75 (0.60, 3.63)	1.93 (0.83, 8.34)	0.553
IFN‐α (pg/mL)	1.54 (0.59, 2.66)	1.09 (0.33, 1.57)	0.352
IFN‐γ (pg/mL)	2.42 (0.81, 6.51)	1.47 (0.31, 6.70)	0.726
TNF‐α (pg/mL)	1.20 (0.50, 2.22)	1.03 (0.35, 1.77)	0.621
IL‐6(pg/mL)	4.00 (2.00, 10.25)	5.00 (4.00, 14.00)	0.247
IL‐8(pg/mL)	16.00 (10.00, 22.25)	22.00 (16.00, 55.00)	0.038*
IL‐10(pg/ml)	5.00 (5.00, 5.00)	5.00 (5.00, 7.00)	0.346
PLT (×10^9^/L)	224.00 (173.75, 277.50)	259.00 (218.00, 328.00)	0.000^#^
PCT (%)	0.24 (0.19, 0.29)	0.28 (0.22, 0.32)	0.001*
MPV (fL)	10.50 (9.90, 11.40)	10.55 (9.58, 11.48)	0.431
PDW (fL)	12.10 (10.80, 14.00)	11.85 (9.80, 14.50)	0.613
P‐LCR (%)	28.90 (23.15, 36.10)	28.50 (20.55, 37.53)	0.420
Lymphocyte counts (cell/μL)	1183.00 (809.00, 1721.00)	1342.50 (1071.50, 2579.25)	0.102
T‐lymphocyte ratio (%)	66.04 ± 12.70	68.79 ± 9.90	0.359
T lymphocyte counts (cell/μL)	733.00 (516.50, 1114.25)	870.50 (738.50, 1512.00)	0.053
Tc/Ts ratio (%)	20.68 (16.05, 30.84)	25.57 (19.89, 34.14)	0.062
Tc/Ts cell counts (cell/μL)	279.50 (145.00, 415.50)	384.50 (261.00, 590.50)	0.013*
Th cell ratio (%)	40.90 ± 12.65	37.81 ± 11.75	0.310
Th cell counts (cell/μL)	441.00 (293.25, 710.25)	551.50 (391.75, 991.25)	0.098
DP cell ratio (%)	0.32 (0.17, 0.51)	0.35 (0.24, 0.81)	0.227
DP cell counts (cell/μL)	4.00 (2.00, 8.00)	5.00 (3.00, 12.75)	0.057
DN cell ratio (%)	1.02 (0.51, 1.95)	1.51 (0.86, 2.63)	0.093
NK cell ratio (%)	13.21 (6.48, 20.46)	13.05 (7.56, 19.55)	0.892
NK cell counts (cell/μL)	126.50 (74.25, 301.25)	212.00 (113.00, 357.00)	0.183
B lymphocyte ratio (%)	15.25 (9.57, 23.05)	13.76 (9.36, 20.39)	0.473
B lymphocyte counts (cell/μL)	152.50 (96.25, 279.75)	201.00 (156.50, 400.00)	0.176
Th/Ts ratio	1.91 (1.30, 3.00)	1.53 (1.04, 2.19)	0.060
IgG (g/L)	12.70 (10.10, 15.10)	13.20 (9.59, 18.40)	0.366
IgA (g/L)	2.13 (1.56, 2.78)	2.45 (1.45, 2.91)	0.606
IgM (g/L)	1.14 (0.77, 1.60)	1.30 (1.15, 1.78)	0.006*
C3 (g/L)	0.98 (0.86, 1.11)	1.02 (0.91, 1.15)	0.077
C4 (g/L)	0.21 (0.17, 0.26)	0.23 (0.17, 0.27)	0.356

DN cell, double negative (CD4‐CD8‐) T‐cells; DP cell, double positive (CD4 + CD8+) T‐cell; IFN, interferon; ILD, interstitial lung disease; MDA5, anti‐melanoma differentiation‐associated gene 5 antibodies; MPV, mean platelet volume; NK cell, natural killer cell; PASP, pulmonary artery systolic pressure; PCT, plateletcrit; PDW, platelet volume distribution width; PE, pulmonary embolism; PH, pulmonary hypertension; P‐LCR, platelet‐large cell ratio; PLT, platelet; Tc/Ts, cytotoxic/suppressor T‐cell; Th cell, helper T‐cell; TNF, tumour necrosis factor.

^#^ *p* < 0.001.

* *p* < 0.05.

### 3.3 Comparison of the ILD‐DM and non‐ILD‐DM characteristics

The ILD‐DM was significantly older than the Non‐ILD‐DM (*p* = 0.019). There were no significant differences in cytokines between these two groups. Additionally, no significant differences in lymphocyte subsets were found. While IgG, IgA and IgM levels were considerably increased in the ILD‐DM than in the Non‐ILD‐DM (*p* = 0.022, 0.002 and 0.029, respectively) (Table 3).

**TABLE 3:** Comparison of the ILD‐DM and non‐ILD‐DM characteristics.

	ILD (*n* = 158)	Non‐ILD (*n* = 214)	*p* value
Age (years)	50 (44, 58)	48.5 (34.75, 58)	0.019*
Gender (male/female)	52/106	72/142	0.882
Ethnicity (Han/Minority)	137/21	183/31	0.743
Smoking (yes/no)	31/127	33/181	0.289
Drinking (yes/no)	22/136	19/195	0.125
Family history (yes/no)	0/158	3/211	0.364
PE (yes/no)	1/157	2/212	1.000
PH (yes/no)	52/58	33/62	0.069
MDA5 (+/−)	25/133	5/209	0.000^#^
PASP (mmHg)	28.00 (21.00, 36.00)	26.00 (21.00, 31.00)	0.072
IL‐1β (pg/mL)	1.28 (0.59, 6.49)	0.98 (0.59, 3.81)	0.507
IL‐2 (pg/mL)	1.38 (0.61, 2.24)	1.06 (0.58, 2.01)	0.258
IL‐4 (pg/mL)	0.87 (0.34, 1.72)	1.18 (0.55, 1.57)	0.366
IL‐5 (pg/mL)	1.24 (0.73, 3.13)	2.22 (1.01, 3.70)	0.157
IL‐12P70 (pg/mL)	0.92 (0.70, 1.82)	0.88 (0.36, 1.60)	0.215
IL‐17 (pg/mL)	2.47 (0.62, 8.51)	1.68 (0.60, 3.17)	0.394
IFN‐α (pg/mL)	1.55 (0.52, 3.14)	1.41 (0.58, 2.35)	0.761
IFN‐γ (pg/mL)	2.46 (0.68, 7.02)	2.11 (1.00, 6.28)	0.774
TNF‐α (pg/mL)	1.21 (0.49, 2.64)	1.12 (0.58, 1.94)	0.691
IL‐6 (pg/mL)	5.00 (3.00, 11.25)	4.00 (2.00, 11.00)	0.229
IL‐8 (pg/mL)	16.00 (11.00, 22.25)	17.00 (10.00, 35.00)	0.935
IL‐10 (pg/mL)	5.00 (5.00, 6.00)	5.00 (5.00, 5.00)	0.579
PLT (×10^9^/L)	233.50 (183.25, 287.25)	225.00 (178.00, 280.00)	0.461
PCT (%)	0.25 (0.20, 0.29)	0.24 (0.19, 0.29)	0.482
MPV (fL)	10.65 (9.80, 11.50)	10.50 (9.80, 11.20)	0.318
PDW (fL)	12.10 (10.53, 14.58)	11.90 (10.65, 13.78)	0.618
P‐LCR (%)	28.80 (22.90, 37.10)	28.90 (23.00, 34.85)	0.399
Lymphocytes counts (cell/μL)	1463.00 (948.00, 1710.00)	1009.50 (717.50, 1953.00)	0.069
T‐lymphocyte ratio (%)	68.54 (60.96, 75.36)	66.03 (55.42, 74.83)	0.428
T lymphocyte counts (cell/μL)	864.50 (612.75, 1128.00)	693.50 (428.75, 1195.50)	0.112
Tc/Ts ratio (%)	23.30 (16.96, 32.06)	20.18 (15.34, 29.62)	0.227
Tc/Ts cell counts (cell/μL)	292.50 (201.50, 489.75)	218.50 (139.75, 429.00)	0.056
Th cell ratio (%)	40.16 ± 12.13	40.79 ± 13.08	0.768
Th cell counts (cell/μL)	457.50 (343.50, 700.75)	460.00 (283.50, 800.00)	0.606
DP cell ratio (%)	0.32 (0.18, 0.45)	0.34 (0.17, 0.60)	0.211
DP cell counts (cell/μL)	4.00 (2.00, 7.25)	4.00 (2.00, 9.25)	0.772
DN cell ratio (%)	1.12 (0.56, 1.91)	1.02 (0.52, 2.36)	0.741
NK cell ratio (%)	13.15 (7.18, 20.15)	12.86 (5.43, 20.46)	0.398
NK cell counts (cell/μL)	194.50 (85.50, 341.00)	112.00 (67.00, 251.50)	0.100
B lymphocyte ratio (%)	13.91 (8.27, 21.17)	16.49 (11.30, 24.49)	0.094
B lymphocyte counts (cell/μL)	160.00 (102.00, 302.25)	183.00 (86.50, 290.50)	0.804
Th/Ts ratio	1.86 (1.18, 2.95)	1.85 (1.50, 2.82)	0.445
IgG (g/L)	13.45 (10.83, 16.18)	12.05 (9.61, 14.88)	0.022*
IgA (g/L)	2.39 (1.63, 3.30)	1.95 (1.53, 2.64)	0.002*
IgM (g/L)	1.25 (0.85, 1.78)	1.15 (0.77, 1.51)	0.029*
C3 (g/L)	1.00 (0.87, 1.10)	0.97 (0.87, 1.13)	0.761
C4 (g/L)	0.22 (0.16, 0.27)	0.21 (0.17, 0.26)	0.647

DN cell, double negative (CD4‐CD8‐) T‐cells; DP cell, double positive (CD4 + CD8+) T‐cell; IFN, interferon; ILD, interstitial lung disease; MDA5, anti‐melanoma differentiation‐associated gene 5 antibodies; MPV, mean platelet volume; NK cell, natural killer cell; PASP, pulmonary artery systolic pressure; PCT, plateletcrit; PDW, platelet volume distribution width; PE, pulmonary embolism; PH, pulmonary hypertension; P‐LCR, platelet‐large cell ratio; PLT, platelet; Tc/Ts, cytotoxic/suppressor T‐cell; Th cell, helper T‐cell; TNF, tumour necrosis factor.

* *p* < 0.05.

^#^ *p* < 0.001.

### 3.4 Comparison of the PH‐DM and non‐PH‐DM characteristics

The PH‐DM was significantly older than Non‐PH‐DM (*p* < 0.001). IL‐17 levels were significantly lower in the PH‐DM (median [IQR]; 0.83 [0.18–1.98] pg/mL) than in the Non‐PH‐DM (median [IQR]; 2.84 [1.15–7.46] pg/mL (*p* = 0.004). While IL‐6 and IL‐10 levels were significantly elevated in the PH‐DM (*p* = 0.008 and 0.001, respectively). In addition, PCT was lower than in the Non‐PH‐DM (*p* = 0.033). Also, double positive (CD4 + CD8+) (DP) cell ratio and B lymphocyte ratio were reduced than in the Non‐PH‐DM (*p* = 0.048 and 0.034, respectively), while natural killer (NK) cell ratio and NK cell counts were significantly elevated than in the Non‐PH‐DM (*p* = 0.013 and 0.030, respectively). Moreover, IgA levels were elevated in the PH‐DM (median [IQR]; 2.34 [1.76–2.86] g/L) than in the Non‐PH‐DM (median [IQR]; 2.07 [1.42–2.78] g/L (*p* = 0.045) (Table 4).

**TABLE 4:** Comparison of the PH‐DM and non‐PH‐DM characteristics.

	PH (*n* = 85)	Non‐PH (*n* = 120)	*p* value
Age (years)	54 (45, 64)	48 (40, 56)	0.000^#^
Gender (Male/Female)	34/51	37/83	0.174
Ethnicity (Han/Minority)	78/7	103/17	0.193
Smoking (yes/no)	20/65	17/103	0.086
Drinking (yes/no)	15/70	12/108	0.111
Family history (yes/no)	0/85	1/119	1.000
ILD (yes/no)	52/33	58/62	0.069
PE (yes/no)	1/84	2/118	1.000
MDA5 (+/−)	8/77	13/107	0.714
IL‐1β (pg/mL)	1.09 (0.48, 3.73)	0.98 (0.59, 5.59)	0.398
IL‐2 (pg/mL)	1.30 (0.72, 2.21)	1.65 (0.56, 2.56)	0.860
IL‐4 (pg/mL)	0.72 (0.36, 1.34)	1.19 (0.58, 1.73)	0.096
IL‐5 (pg/mL)	1.56 (0.72, 3.22)	2.41 (1.23, 3.87)	0.102
IL‐12P70 (pg/mL)	0.94 (0.40, 1.58)	0.98 (0.70, 2.00)	0.836
IL‐17 (pg/mL)	0.83 (0.18, 1.98)	2.84 (1.15, 7.46)	0.004*
INF‐α (pg/mL)	1.09 (0.51, 2.02)	1.67 (0.82, 3.65)	0.168
INF‐γ (pg/mL)	2.07 (0.87, 5.02)	3.18 (1.10, 10.05)	0.129
TNF‐α (pg/mL)	0.81 (0.36, 2.01)	1.51 (0.58, 2.54)	0.344
IL‐6 (pg/mL)	10.00 (3.00, 15.50)	3.50 (2.00, 5.00)	0.008*
IL‐8 (pg/mL)	19.00 (12.50, 44.50)	16.50 (10.00, 24.50)	0.317
IL‐10 (pg/mL)	5.00 (5.00, 7.50)	5.00 (5.00, 5.00)	0.001*
PLT (×10^9^/L)	225.00 (167.00, 280.00)	242.50 (194.75, 298.25)	0.069
PCT (%)	0.23 (0.19, 0.29)	0.26 (0.21, 0.30)	0.033*
MPV (fL)	10.60 (9.80, 11.30)	10.30 (9.80, 11.23)	0.738
PDW (fL)	12.05 (10.50, 13.75)	11.70 (10.60, 13.85)	0.943
P‐LCR (%)	28.80 (22.38, 35.12)	27.10 (23.08, 35.32)	0.857
Lymphocyte counts (cell/μL)	1227.50 (961.50, 1904.00)	1488.00 (823.00, 1831.00)	0.622
T‐lymphocyte ratio (%)	67.79 (56.69, 74.85)	69.66 (60.22, 76.31)	0.389
T lymphocyte counts (cell/μL)	825.00 (618.50, 1153.00)	826.00 (545.00, 1240.00)	0.907
Tc/Ts ratio (%)	23.61 (15.56, 35.76)	22.99 (18.75, 31.54)	0.949
Tc/Ts cell counts (cell/μL)	293.00 (195.50, 496.50)	282.00 (167.00, 501.00)	0.687
Th cell ratio (%)	37.03 ± 12.36	41.87 ± 12.53	0.068
Th cell counts (cell/μL)	452.00 (319.00, 733.00)	480.00 (356.00, 730.00)	0.423
DP cell ratio (%)	0.24 (0.16, 0.45)	0.37 (0.20, 0.79)	0.048*
DP cell counts (cell/μL)	3.00 (2.00, 7.00)	4.00 (2.00, 12.00)	0.279
DN cell ratio (%)	1.18 (0.55, 2.34)	1.04 (0.48, 2.37)	0.946
NK cell ratio (%)	16.04 (9.65, 27.04)	11.90 (5.72, 18.09)	0.013*
NK cell counts (cell/μL)	246.50 (107.75, 396.75)	138.00 (66.50, 293.00)	0.030*
B lymphocyte ratio (%)	10.69 (6.25, 18.72)	13.91 (10.53, 22.45)	0.034*
B lymphocyte counts (cell/μL)	136.00 (68.50, 289.75)	169.00 (105.00, 312.50)	0.163
Th/Ts ratio	1.79 (0.98, 2.97)	1.88 (1.30, 2.62)	0.910
IgG (g/L)	13.20 (11.00, 15.80)	12.60 (9.67, 15.00)	0.226
IgA (g/L)	2.34 (1.76, 2.86)	2.07 (1.42, 2.78)	0.045*
IgM (g/L)	1.11 (0.81, 1.55)	1.28 (0.83, 1.73)	0.244
C3 (g/L)	0.94 (0.86, 1.08)	1.00 (0.87, 1.14)	0.085
C4 (g/L)	0.20 (0.16, 0.25)	0.22 (0.17, 0.27)	0.208

DN cell, double negative (CD4‐CD8‐) T‐cells; DP cell, double positive (CD4 + CD8+) T‐cell; IFN, interferon; ILD, interstitial lung disease; MDA5, anti‐melanoma differentiation‐associated gene 5 antibodies; MPV, mean platelet volume; NK cell, natural killer cell; PCT, plateletcrit; PDW, platelet volume distribution width; PE, pulmonary embolism; PH, pulmonary hypertension; P‐LCR, platelet‐large cell ratio; PLT, platelet; Tc/Ts, cytotoxic/suppressor T‐cell; Th cell, helper T‐cell; TNF, tumour necrosis factor.

^#^ *p* < 0.001.

* *p* < 0.05.

It is confirmed that PH is a common complication of ILD.^3^ Analyses in subgroup were performed to exclude the effect of ILD on PH (Table 5). In the ILD‐DM subgroup analysis, the Han population had a higher proportion of PH than that of Non‐PH (*p* = 0.024). In addition, IL‐6 and IL‐10 levels were considerably higher than in Non‐PH (*p* = 0.004 and 0.039, respectively). Moreover, helper T (Th) cell ratio, Th cell counts, DP cell ratio, B lymphocyte ratio and B lymphocyte counts were lower in PH than in Non‐PH (*p* = 0.027, 0.044, 0.031, 0.045 and 0.029, respectively). In contrast, the NK cell ratio in PH was significantly increased than in Non‐PH. Besides, the C4 was lower in PH than in Non‐PH (0.20 ± 0.08 g/L vs. 0.24 ± 0.09 g/L *p* = 0.041). In the Non‐ILD‐DM subgroup analysis, PH was significantly older than Non‐PH (*p* = 0.001). More importantly, IL‐17 and interferon (IFN) ‐γ were significantly lower in PH than in Non‐PH (*p* = 0.026 and 0.043, respectively). Also, the PCT was lower in PH than in Non‐PH (*p* = 0.031). However, lymphocyte subsets in this subgroup were no differences. Additionally, IgG levels were considerably elevated in PH (median [IQR]; 13.35 [10.83–16.28] g/L) than in Non‐PH (median [IQR]; 11.70 [9.12–13.70] g/L (*p* = 0.016).

**TABLE 5:** Comparison of the ILD‐DM subgroup and non‐ILD‐DM subgroup characteristics.

	ILD			Non‐ILD		
	PH (*n* = 52)	Non‐PH (*n* = 58)	*p* value	PH (*n* = 33)	Non‐PH (*n* = 62)	*p* value
Age (years)	53 (46, 63)	50.5 (44, 57)	0.125	54.18 ± 15.34	41.90 ± 16.49	0.001*
Gender (male/female)	21/31	19/39	0.406	13/20	18/44	0.305
Ethnicity (Han/Minority)	50/2	48/10	0.024*	28/5	55/7	0.830
Smoking (yes/no)	13/39	11/47	0.444	7/26	6/56	0.213
Drinking (yes/no)	11/41	6/52	0.117	4/29	6/56	0.985
Family history (yes/no)	0/52	0/58	―	0/33	1/61	1.000
PE (yes/no)	1/51	0/58	0.956	0/33	2/60	0.770
MDA5 (+/−)	8/44	12/46	0.471	0/33	1/61	1.000
IL‐1β (pg/mL)	3.73 (1.14, 5.20)	1.06 (0.59, 6.90)	0.521	0.50 (0.44, 1.09)	0.98 (0.64, 4.88)	0.091
IL‐2 (pg/mL)	1.30 (0.92, 2.51)	1.89 (0.68, 2.72)	0.594	1.09 (0.63, 2.02)	1.02 (0.52, 2.74)	0.842
IL‐4 (pg/mL)	0.72 (0.28, 1.56)	1.19 (0.64, 1.93)	0.274	0.88 ± 0.52	1.19 ± 0.60	0.226
IL‐5 (pg/mL)	0.74 (0.42, 3.58)	1.54 (1.08, 3.32)	0.503	1.79 ± 1.17	3.35 ± 2.22	0.097
IL‐12P70 (pg/mL)	1.5 (0.90, 2.62)	0.91 (0.71, 2.24)	0.467	0.44 (0.29, 1.39)	1.05 (0.64, 1.67)	0.298
IL‐17 (pg/mL)	0.71 (0.18, 8.43)	3.13 (0.89, 9.79)	0.127	0.86 (0.16, 1.93)	2.57 (1.24, 6.68)	0.026*
IFN‐α (pg/mL)	1.37 (0.23, 2.28)	1.88 (0.98, 4.72)	0.237	0.92 (0.60, 1.99)	1.39 (0.50, 2.86)	0.528
IFN‐γ (pg/mL)	4.75 (0.87, 6.65)	2.84 (0.67, 12.24)	0.871	1.83 (0.62, 3.29)	6.22 (1.82, 10.05)	0.043*
TNF‐α (pg/mL)	1.58 ± 1.19	1.63 ± 1.14	0.933	0.79 (0.36, 1.03)	1.65 (0.40, 2.76)	0.278
IL‐6 (pg/mL)	10.00 (5.75, 17.50)	4.00 (2.00, 5.00)	0.004*	4.00 (2.00, 13.00)	3.00 (2.00, 5.75)	0.574
IL‐8 (pg/mL)	22.00 (15.50, 40.75)	16.00 (11.75, 21.50)	0.094	14.00 (5.00, 73.00)	17.00 (7.50, 96.00)	0.836
IL‐10 (pg/mL)	5.50 (5.00, 7.25)	5.00 (5.00, 5.00)	0.039*	5.00 (5.00, 10.00)	5.00 (5.00, 5.00)	0.357
PLT (×10^9^/L)	232.00 (181.00, 285.00)	243.00 (195.50, 297.50)	0.306	203.50 (166.25, 272.75)	242.00 (190.00, 303.00)	0.078
PCT (%)	0.25 (0.18, 0.29)	0.27 (0.21, 0.30)	0.269	0.21 (0.19, 0.29)	0.25 (0.22, 0.30)	0.031*
MPV (fL)	10.70 (9.80, 11.43)	10.30 (9.80, 11.40)	0.559	10.45 (9.53, 11.00)	10.30 (9.90, 10.90)	0.690
PDW (fL)	12.15 (10.70, 14.05)	11.50 (10.20, 14.70)	0.410	11.55 (10.10, 13.18)	11.90 (11.10, 13.40)	0.295
P‐LCR (%)	28.95 (22.83, 36.15)	26.60 (22.30, 36.40)	0.592	27.70 (20.48, 32.43)	27.50 (23.80, 32.90)	0.583
Lymphocyte counts (cell/μL)	1193.00 (939.50, 1673.50)	1505.00 (950.00, 1831.00)	0.284	1317.00 (907.50, 2555.50)	978.00 (641.75, 1883.00)	0.102
T‐lymphocyte ratio (%)	64.16 ± 13.38	66.93 ± 11.23	0.394	67.46 ± 14.00	69.41 ± 12.82	0.668
T lymphocyte counts (cell/μL)	825.00 (609.00, 1064.00)	937.50 (595.00, 1140.25)	0.437	831.00 (651.75, 1291.75)	761.00 (461.00, 1240.00)	0.506
Tc/Ts ratio (%)	23.73 (16.10, 39.53)	22.62 (17.30, 31.63)	0.641	20.43 (13.87, 33.31)	24.03 (18.90, 30.39)	0.610
Tc/Ts cell counts (cell/μL)	291.00 (186.00, 479.00)	322.00 (199.75, 501.75)	0.864	339.50 (204.50, 535.00)	243.00 (148.00, 482.00)	0.394
Th cell ratio (%)	34.64 ± 12.81	41.49 ± 10.20	0.027*	40.95 ± 10.92	42.45 ± 15.75	0.756
Th cell counts (cell/μL)	410.00 (272.00, 607.00)	520.50 (386.50, 721.50)	0.044*	653.00 (442.50, 824.50)	466.00 (294.00, 797.00)	0.244
DP cell ratio (%)	0.21 (0.12, 0.40)	0.36 (0.20, 0.77)	0.031*	0.34 (0.17, 0.62)	0.40 (0.16, 1.00)	0.610
DP cell counts (cell/μL)	3.00 (2.00, 6.00)	4.50 (2.00, 12.00)	0.104	5.50 (3.00, 10.75)	4.00 (2.00, 13.00)	0.841
DN cell ratio (%)	1.12 (0.53, 1.91)	0.87 (0.45, 1.89)	0.874	1.44 (0.60, 3.00)	1.67 (0.55, 3.04)	0.986
NK cell ratio (%)	17.83 (10.65, 28.20)	12.78 (6.27, 18.25)	0.019*	13.36 (7.38, 24.45)	9.26 (4.74, 18.18)	0.276
NK cell counts (cell/μL)	255.00 (113.00, 371.00)	211.00 (59.75, 351.50)	0.161	221.00 (77.00, 479.50)	95.00 (72.00, 185.50)	0.169
B lymphocyte ratio (%)	9.43 (6.19, 18.08)	14.16 (9.95, 22.35)	0.045*	12.99 (6.03, 20.49)	13.91 (11.10, 22.95)	0.362
B lymphocyte counts (cell/μL)	110.00 (75.00, 198.50)	170.00 (120.25, 313.25)	0.029*	211.00 (59.50, 383.00)	120.00 (89.50, 315.50)	0.972
Th/Ts ratio	1.71 (0.94, 3.32)	1.76 (1.22, 2.58)	0.834	2.07 ± 0.96	2.14 ± 1.04	0.820
IgG (g/L)	12.90 (11.00, 15.30)	14.05 (10.80, 17.15)	0.415	13.35 (10.83, 16.28)	11.70 (9.12, 13.70)	0.016*
IgA (g/L)	2.53 (2.02, 3.29)	2.31 (1.59, 3.29)	0.305	2.10 (1.65, 2.69)	1.83 (1.32, 2.48)	0.158
IgM (g/L)	1.10 (0.81, 1.84)	1.39 (0.85, 1.95)	0.162	1.15 (0.68, 1.49)	1.19 (0.76, 1.66)	0.690
C3 (g/L)	1.01 (0.87, 1.08)	1.02 (0.85, 1.15)	0.387	0.90 (0.84, 1.05)	1.00 (0.89, 1.13)	0.065
C4 (g/L)	0.20 ± 0.08	0.24 ± 0.09	0.041*	0.22 (0.16, 0.26)	0.20 (0.17, 0.27)	0.993

DN cell, double negative (CD4‐CD8‐) T‐cells; DP cell, double positive (CD4 + CD8+) T‐cell; IFN, interferon; ILD, interstitial lung disease; MDA5, anti‐melanoma differentiation‐associated gene 5 antibodies; MPV, mean platelet volume; NK cell, natural killer cell; PCT, plateletcrit; PDW, platelet volume distribution width; PE, pulmonary embolism; PH, pulmonary hypertension; P‐LCR, platelet‐large cell ratio; PLT, platelet; Tc/Ts, cytotoxic/suppressor T‐cell; Th cell, helper T‐cell; TNF, tumour necrosis factor.

* *p* < 0.05.

### 3.5 Comparison of the MDA5^+^ DM and MDA5^−^ DM characteristics

The proportion of males with the MDA5^+^ DM was higher than that with the MDA5^−^ DM (*p* = 0.043). Also, the MDA5^+^ DM owned more smoking history than the MDA5^−^ DM (*p* = 0.003). The MDA5^+^ DM had a higher rate of combining ILD than the MDA5^−^ DM (*p* < 0.001). Moreover, NK cell ratio and NK cell counts were significantly lower than in the MDA5^−^ DM (*p* = 0.004 and 0.002, respectively). Furthermore, IgA and C4 levels were considerably higher than in the MDA5^−^ DM (*p* = 0.014 and 0.005, respectively) (Table 6).

**TABLE 6:** Comparison of the MDA5^+^ DM and MDA5^−^ DM characteristics.

	MDA5^+^ (*n* = 30)	MDA5^−^ (*n* = 342)	*p* value
Age (years)	49.5 (41.5, 56)	49 (40, 58.25)	0.592
Gender (Male/Female)	15/15	109/233	0.043*
Ethnicity (Han/Minority)	27/3	293/49	0.703
Smoking (yes/no)	11/19	53/289	0.003*
Drinking (yes/no)	6/24	35/307	0.182
Family history (yes/no)	0/30	3/339	1.000
ILD (yes/no))	25/5	133/209	0.000^#^
PE (yes/no)	0/30	3/339	1.000
PH (yes/no)	8/13	77/107	0.741
PASP (mm Hg)	24.00 (21.00, 30.00)	28.00 (21.00, 34.00)	0.364
IL‐1β (pg/mL)	1.22 (0.60, 15.59)	1.06 (0.59, 3.82)	0.559
IL‐2 (pg/mL)	1.45 (0.59, 3.18)	1.20 (0.58, 2.14)	0.773
IL‐4 (pg/mL)	0.48 (0.29, 1.65)	1.12 (0.55, 1.67)	0.179
IL‐5 (pg/mL)	1.95 (0.68, 3.50)	1.86 (0.98, 3.29)	0.821
IL‐12P70 (pg/mL)	0.69 (0.28, 1.35)	0.94 (0.51, 1.71)	0.268
IL‐17 (pg/mL)	1.34 (0.70, 5.61)	1.78 (0.59, 3.64)	0.797
IFN‐α (pg/mL)	1.09 (0.10, 5.33)	1.55 (0.59, 2.52)	0.438
IFN‐γ (pg/mL)	0.69 (0.31, 13.23)	2.43 (1.07, 6.51)	0.258
TNF‐α (pg/mL)	1.21 (0.47, 2.56)	1.16 (0.52, 2.00)	0.875
PLT (×10^9^/L)	231.00 (189.50, 264.50)	231.00 (178.75, 289.25)	0.672
PCT (%)	0.23 (0.19, 0.27)	0.24 (0.20, 0.30)	0.427
MPV (fL)	10.30 (9.90, 11.05)	10.60 (9.80, 11.40)	0.438
PDW (fL)	10.90 (10.10, 13.00)	12.10 (10.80, 14.20)	0.063
P‐LCR (%)	25.80 (22.70, 33.25)	29.15 (22.98, 36.60)	0.305
Lymphocyte counts (cell/μL)	978.00 (823.00, 1542.00)	1307.00 (852.75, 1904.00)	0.111
T‐lymphocyte ratio (%)	72.50 ± 9.17	65.35 ± 12.54	0.140
T lymphocyte counts (cell/μL)	795.00 (553.50, 992.00)	771.00 (532.00, 1169.00)	0.720
Tc/Ts ratio (%)	23.61 (17.22, 36.18)	22.54 (16.03, 31.07)	0.406
Tc/Ts cell counts (cell/μL)	281.00 (163.00, 393.50)	282.00 (159.00, 501.00)	0.678
Th cell ratio (%)	44.20 ± 10.96	39.77 ± 12.72	0.137
Th cell counts (cell/μL)	438.00 (340.50, 560.50)	466.00 (294.00, 751.00)	0.676
DP cell ratio (%)	0.29 (0.12, 0.39)	0.32 (0.18, 0.60)	0.172
DP cell counts (cell/μL)	2.00 (2.00, 4.50)	4.00 (2.00, 9.31)	0.051
DN cell ratio (%)	1.69 (0.74, 2.58)	1.01 (0.53, 1.92)	0.191
NK cell ratio (%)	6.32 (5.12, 14.35)	13.67 (9.15, 21.79)	0.004*
NK cell counts (cell/μL)	79.00 (52.00, 109.00)	175.50 (77.75, 346.50)	0.002*
B lymphocyte ratio (%)	16.94 (9.82, 21.35)	14.05 (9.43, 23.04)	0.604
B lymphocyte counts (cell/μL)	153.00 (120.00, 276.00)	171.00 (96.75, 296.75)	0.921
Th/Ts ratio	2.02 (1.04, 2.82)	1.85 (1.24, 2.94)	0.791
IgG (g/L)	12.70 (9.57, 14.15)	12.70 (10.10, 15.30)	0.680
IgA (g/L)	2.48 (2.05, 3.47)	2.12 (1.53, 2.77)	0.014*
IgM (g/L)	1.27 (0.86, 1.60)	1.16 (0.80, 1.63)	0.502
C3 (g/L)	1.03 (0.82, 1.16)	0.98 (0.87, 1.11)	0.682
C4 (g/L)	0.25 (0.21, 0.30)	0.21 (0.16, 0.26)	0.005*

DN cell, double negative (CD4‐CD8‐) T‐cells; DP cell, double positive (CD4 + CD8+) T‐cell; IFN, interferon; ILD, interstitial lung disease; MDA5, anti‐melanoma differentiation‐associated gene 5 antibodies; MPV, mean platelet volume; NK cell, natural killer cell; PCT, plateletcrit; PDW, platelet volume distribution width; PE, pulmonary embolism; PH, pulmonary hypertension; P‐LCR, platelet‐large cell ratio; PLT, platelet; Tc/Ts, cytotoxic/suppressor T‐cell; Th cell, helper T‐cell; TNF, tumour necrosis factor.

* *p* < 0.05.

^#^ *p* < 0.001.

## 4 DISCUSSION

The findings of this study revealed that the age of the Han population with DM patients was older than that of the ethnic minority, while PLT, PCT, IL‐8 and IgM were lower in the Han population. In addition, IgG, IgA and IgM levels in the ILD‐DM were higher than in the Non‐ILD‐DM. Moreover, IL‐6 and IL‐10 were increased in the PH‐DM than in the Non‐PH‐DM, while PCT, IL‐17, DP cell ratio and B lymphocyte ratio were reduced in the PH‐DM. Meanwhile, the MDA5^+^ DM had more smoking history than the MDA5^−^ DM and the incidence of ILD was higher in the MDA5^+^ DM. Also, the MDA5^+^ DM had higher levels of IgA and C4, while the MDA5^+^ DM had lower levels of NK cell ratio and NK cell counts.

The result in the study showed that DM patients of the Han population were older than that of the ethnic minority, indicating that the age of clinical onset of DM in the ethnic minority tended to be younger. In addition, IL‐8 and IgM were lower in patients with DM in the Han population than in the minority population. It is well known that IL‐8 is a chemokine that plays an important role in most inflammatory conditions,^12^ while IgM suppresses excessive inflammatory responses mediated by autoimmune mechanisms.^13^ However, what exactly are the reasons for the differences between IL‐8 and IgM among ethnic groups need more research in the future. Meanwhile, PLT and PCT were lower than that of the ethnic minority in this study, which suggested that it was more important to pay attention to the changes of PLT and PCT in the Han population DM patients in clinical practice. Moreover, ethnicity‐related T lymphocyte differences have not been reported in DM patients. Our study showed that Tc/Ts counts in the Han population were significantly lower than in the ethnic minority DM patients, which suggested that Tc/Ts counts might be different among ethnicity in China.

ILD, a common complication of DM, has a significant impact on prognosis.^1^ It is shown that advanced age is an independent risk factor for ILD in DM patients.^14^ Our study also showed that the age of the ILD‐DM was higher than that of the Non‐ILD‐DM.

It is reported that older age group is more likely suffer from PH, which means that there is a correlation between PH and age.^15^ Our study showed that PH‐DM patients were significantly older than Non‐PH‐DM patients. However, whether the correlation between PH and age is interfered with by DM is unclear and more researches are needed. It has been reported that IL‐6 acts as both a proinflammatory and an anti‐inflammatory cytokine and was associated with excessive cell proliferation and pulmonary vascular remodelling.^8^, ^16^ Moreover, elevated IL‐6 levels have been demonstrated in DM and in PH, where it played a major role in the inflammatory process of DM and in the pathogenesis of PH.^16^, ^17^ The same results were obtained in this study. IL‐10 is one of the most important anti‐inflammatory cytokines that inhibit excessive inflammatory processes.^17^ Elevated levels of IL‐10 were found in patients with PH, which might be a counter‐regulatory mechanism of lung tissue inflammation.^17^ Therefore, the elevated IL‐10 level in PH‐DM patients in our study might also be related to the anti‐regulatory mechanism of lung tissue inflammation. It has been presented that IL‐17 was highly expressed in both DM and PH and this elevation was also associated with the pathogenesis of DM.^18^, ^19^ Contrary to the findings of other researches, our study found that IL‐17 was significantly lower in PH than in Non‐PH, both in the overall analysis and in the subgroup analysis. We speculated that such opposite findings might be due to differences in the characteristics or ethnicity of the study populations. It has been reported that PLT activation played an important role in the pathogenesis of PH, and PCT was a marker of PLT activation.^6^, ^20^ Our study found that PH had lower PCT than Non‐PH, which indicated that PCT might be linked to the aetiology of PH in DM patients. Also, some studies suggested that T and B lymphocytes might be intimately associated with the development of PH.^21^ In this study, DP cells and B lymphocytes were shown to be considerably lower in PH than in Non‐PH, and differences in these indicators were also seen in the subgroup analysis of the ILD‐DM. Besides that, it has been reported that peripheral blood T lymphocytopenia was a prevalent clinical phenomenon in DM and its deficiency also increased the tendency to develop PH in animal experiments.^22^, ^23^ Therefore, we speculated that PH might be caused by T lymphocytopenia in DM. Meanwhile, it has been suggested that the decrease in B lymphocytes might be due to the accumulation of B lymphocytes in inflamed muscle tissue in patients with DM, which caused B lymphocytes to decrease in the peripheral blood.^24^ Since B lymphocytes were also implicated in the pathogenesis of PH, we hypothesized that the incidence of PH might also be connected to the decline in peripheral B lymphocytes in DM patients. Previous reports have shown that NK cells were reduced in PH alone.^22^ However, our study showed that NK cells were considerably higher in PH than in Non‐PH, suggesting that a possible difference in the pathogenesis between PH caused by DM and PH alone. Finally, our study showed that IgA levels were significantly higher in the PH‐DM than in the Non‐PH‐DM, which seemed to suggest that IgA was involved in the aetiology of PH‐DM. However, we performed subgroup analysis and found that there was no difference in IgA levels between PH and Non‐PH in both the ILD‐DM and the Non‐ILD‐DM subgroups. Therefore, we made the assumption that IgA might not be a major factor causing PH in DM patients.

Currently, there are contradictory perspectives on whether there is an association between MDA5 and gender in patients with DM. Research in China reported no correlation between the incidence of anti‐MDA5 antibodies and the patients' gender,^25^ while a study in Europe concluded that patients with MDA5^+^ DM were mainly female.^11^ Our study showed that the proportion of male with the MDA5^+^ DM was higher than that with the MDA5^−^ DM. It seemed that race might have an influence on the connection between MDA5 and gender. In addition, there are no research studies that demonstrate a correlation between smoking and MDA5^+^ DM. Our study showed that the MDA5^+^ DM had more smoking history than the MDA5^−^ DM. Nevertheless, due to the small number of smokers we included, further research is needed to explain whether smoking is related to MDA5^+^ DM. A growing number of studies have shown that one of the typical features of MDA5^+^ DM was the presence of ILD.^26^ Anti‐ MDA5 antibodies might also help with the diagnosis of ILD when found in DM.^26^ Moreover, it has been reported that peripheral lymphopenia was prevalent and easily detected in MDA5^+^ DM‐ILD.^27^ Our study also found that NK cells were significantly lower in the MDA5^+^ DM and the changes might be due to the transfer of lymphocytes to the lungs to participate in the local immune response.^27^ Also, it has been shown that anti‐MDA5 antibodies in serum were mainly present in the form of IgA and other forms.^28^ Because IgA levels in our research were considerably higher in the MDA5^+^ DM than in the MDA5^−^ DM, we hypothesized that anti‐MDA5 antibodies in MDA5^+^ DM might be present in the form of IgA. In this study, C4 levels were significantly higher in the MDA5^+^ DM than in the MDA5^−^ DM, suggesting that elevated C4 levels might be associated with anti‐MDA5 antibodies in DM, which was consistent with other findings.^29^

This study has some limitations. First, this study was a small, retrospective study with limited case data collected. Also, being a non‐multicentre study, only DM cases from one hospital were included, and the possibility of unintentional selection bias could not be completely excluded. Therefore, prospective studies with large sample sizes are needed to confirm our conclusions in future. Secondly, data related to the prognosis of patients with DM were not collected in this study and the findings were not compared with clinical long‐term prognosis. Thus, not allowing further assessment of whether the influencing factors included had an impact on disease progression and outcome in patients with DM. In addition, the age difference between the Han population and the Minority population may be a confounding factor for the study, so the differences of other parameters such as Tc/Ts cell counts between the two populations may also be due to age. Accordingly, future research is needed on the relationship between ethnicity and other factors and to exclude the confounding factor of age. Indeed, differences in immunoglobulins between the two groups may be influenced by Sjogren syndrome (SS). However, because some patients in our study were not tested for SS‐specific antibodies, the diagnosis of SS was failed to be excluded from the collection of medical records. Accordingly, the subsequent research needs to exclude the effect of SS on DM. Finally, no correlation analysis was performed in this study, and it was not possible to grasp the correlation and closeness between the clinical features we studied and ethnicity, PH, ILD and MDA5^+^ in DM patients, respectively.

## 5 CONCLUSION

Han population is older than ethnic minority in China for DM patients and has lower levels of IL‐8 and IgM than ethnic minority. Also, ILD‐DM has higher levels of IgG, IgM and IgA than that of Non‐ILD‐DM. Additionally, PH‐DM has higher levels of IL‐6, IL‐10 and lower levels of IL‐17 than that of Non‐PH‐DM. Meanwhile, it is possible that the decrease in DP cell ratio and B lymphocyte ratio are associated with the development of PH in DM. Moreover, MDA5^+^ DM is more likely to have combined ILD. Anti‐MDA5 antibodies in MDA5^+^ DM may be present in the form of IgA and high levels of C4 may be associated with anti‐MDA5 antibodies.

## AUTHOR CONTRIBUTIONS

Chulin Wang designed and performed the research, collected and analysed the data, and wrote the paper. Han Yang collected and analysed the data and wrote the paper. Tongfen Li designed the research and wrote the paper. Yiqiong Wen designed the research and collected the data. Heran Yang collected and analysed the data. Weifeng Tang performed the research and analysed the data. Lin Li designed and performed research. Shibo Sun designed and performed the research, analysed the data, and wrote the paper.

## CONFLICT OF INTEREST STATEMENT

The authors declare that they have no conflict of interest, and the manuscript is approved by all authors for publication.

### ETHICS STATEMENT

This study was approved by Ethics Committee of the First Affiliated Hospital Kunming Medical University and conformed to the Declaration of Helsinki.

## Data Availability

The data of the present study are available from the corresponding author on reasonable request.
